# The role, challenges, and impact of occupational therapists in custodial settings: A scoping review

**DOI:** 10.1111/1440-1630.70042

**Published:** 2025-08-07

**Authors:** Elizabeth Elder, Shannon Werner, Julia Crilly

**Affiliations:** ^1^ School of Nursing and Midwifery Griffith University Gold Coast Queensland Australia; ^2^ Department of Emergency Medicine Gold Coast Health Gold Coast Queensland Australia; ^3^ Centre for Work, Organisation and Wellbeing Gold Coast Queensland Australia; ^4^ Department of Occupational Therapy Princess Alexandra Hospital, Metro South Health Brisbane Queensland Australia; ^5^ Centre for Mental Health Griffith University Gold Coast Queensland Australia

**Keywords:** custodial settings, healthcare, jail, occupational therapy, prison, review, social determinant of health

## Abstract

**Introduction:**

The number people in custodial settings (i.e., jails and prisons) is increasing globally. In addition to detention and supervision, rehabilitation and skill development are often key goals of corrective services. The high care needs and vulnerability of detainees can often make this challenging, requiring input from a variety of health‐care professionals, including occupational therapists. The aim of this scoping review was to synthesise the peer‐reviewed literature regarding the role of occupational therapists in custodial settings, the challenges occupational therapists experience in custodial settings, and the impact of occupational therapy on outcomes for people in custody.

**Methods:**

A scoping review guided by the Joanna Briggs Institute approach was undertaken. Three online databases (CINAHL, EMBASE, and the Criminal Justice Database [PROQUEST]) were searched from January 2013 to May 2024. Studies were included if the Population was occupational therapist(s), the Context was a custodial setting (jail/prison/correctional facility), and the Concept was the role of, challenges to, and the outcome/s of occupational therapy. The Mixed Methods Assessment Tool was used to appraise study quality.

**Consumer and Community Involvement:**

There was no consumer or community involvement.

**Results:**

Eleven studies were included in this review; three mixed methods studies, one participatory research design, and one critical reflection, with the quality of evidence varying. The role of occupational therapists was primarily in providing direct care. Challenges experienced by occupational therapists were classified into four categories: (i) engagement, (ii) environmental, (iii) governance, and (iv) resourcing. Acts of violence and recidivism rates decreased in detainees who received occupational therapy.

**Conclusion:**

Despite the often‐restrictive nature of jails and prisons, occupational therapists working in custodial settings are in a unique position to develop and implement interventions that support detainees with developing life skills and improve post incarceration employment opportunities. This in turn is suggested to contribute to reduced recidivism.

**PLAIN LANGUAGE SUMMARY:**

People held in prisons and jails often have health and social care needs. Not meeting these needs can lead to housing and job problems after being released. Occupational therapists have an important job. Little is known about what occupational therapists do in custodial settings. This review sought to understand this. Findings showed that staff and detainees liked having occupational therapists. They helped with managing the detainees' feelings and reduce anger. They also helped detainees to get a job and housing after being released.

**Key Points for Occupational Therapy**

- Occupational therapists are uniquely placed to address some of the social determinants of health for detainees.
- Occupational therapy while in custody has contributed to reduced rates of recidivism.
- Moving to a salutogenic model of detainee care has benefits both during and post incarceration.

## 1 INTRODUCTION

Custodial settings include, but are not limited to, prisons, jails, forensic psychiatric hospitals, juvenile detention centres, or community re‐entry facilities (Farnworth & Muñoz, 2009). The number of people detained in custody (i.e., jails/prison) has increased globally over the past few decades (Australian Institute of Health and Welfare, 2022a; Fair & Walmsley, 2024; Kang‐Brown et al., 2021; Sturge, 2021). Since the year 2000, the world prison population has increased 27%, and in 2021, there were nearly 11 million prisoners throughout the world (Fair & Walmsley, 2024). Prison population rates and growth vary within and between continents (Fair & Walmsley, 2024). In Australia, for example, the prison population has risen by 93% since 2000 from 21,714 to 41,929 in 2023, in the United Kingdom (UK) (England and Wales) the prison population has increased 36% since 2000 from 64,602 to 87,699 in 2024, whereas in South Africa the prison population has decreased 8% since 2000 from 171,462 to 157,056 in 2023 and in Singapore the prison population has decreased 45% since 2000 from 13,791 to 9536 in 2022 (Fair & Walmsley, 2024). In the United States of America (USA), the prison population has decreased slightly by 9% since 2000 from 1,937,482 to 1,767,200 in 2021 (Fair & Walmsley, 2024).

In jurisdictions such as Australia and the UK, prisoners diagnosed with significant mental health conditions and where the protection of the wider community is considered justified, are likely to be admitted to a forensic mental health facility for either part of, or for the duration of their incarceration period (Hare Duke et al., 2018; Huang et al., 2023). Internationally, the number of psychiatric beds allocated to forensic services has risen in recent decades (Tully et al., 2024) although this varies between countries (Tomlin et al., 2021); for example, from 1 bed per 100,000 in Switzerland to 12 beds per 100,000 in the Netherlands (Chow & Priebe, 2016). Furthermore, the varied models of care and lack of consistency in standards with forensic mental health services has been criticised (Kennedy et al., 2019). Depending on the setting that they are detained, justice involved people can be referred to as ‘detainees’ or ‘remanded prisoners’ (if detained awaiting trial/sentencing), ‘inmates’ or ‘prisoners’ (if on trial or sentenced and in prison) (Yoon et al., 2017) or ‘forensic patients’ if detained within a forensic medical facility (Mullen et al., 2022). For this review, individuals held within short‐term, or long‐term custodial facilities or forensic medical facilities are referred to as ‘detainees’.

People detained in custodial settings are an especially vulnerable population often with high health‐care needs (Australian Institute of Health and Welfare, 2020; McLeod et al., 2020). The social determinants of health experienced by detainees in custodial settings is often worse than that of the general population (Kinner & Butler, 2017; Nishar et al., 2023). For example, mental health concerns and substance misuse disorders are often over‐represented among the custodial population (Butler et al., 2011; Young et al., 2018). Furthermore, the unique and complex needs of people in custodial settings can make providing individualised, meaningful, and targeted care challenging (Ismail & de Viggiani, 2017; Leonard, 2004; Tavoschi et al., 2019) especially when overlayed with security and safety considerations.

Much of the literature around health‐care delivery in short‐term custodial settings (Wardrop et al., 2021), prisons (Karaaslan & Aslan, 2019; Pont & Harding, 2019; Ronco, 2021; Wong et al., 2018), and forensic mental health facilities (Dean, 2020; Maruca & Shelton, 2016; McKenna & Sweetman, 2021) tends to focus on the role of doctors/psychiatrists and/or nurses, with limited descriptions of other allied health professionals such as physiotherapists, social workers, or occupational therapists. However, the complex and restrictive nature of custodial settings, presents unique challenges, including social isolation (Kyprianides & Easterbrook, 2020), as well as limited access to rehabilitative and meaningful activities (Edwards, 2021)—areas occupational therapists are ideally positioned to assist with. Central to their role, occupational therapists facilitate engagement in meaningful activities, such as socialising, employment, rehabilitation, and activities of daily living (Di Tommaso et al., 2016), areas crucial in reducing recidivism (Ramakers et al., 2017) and improving detainee wellbeing (Baybutt & Chemlal, 2016; Esposito, 2015). Additionally, they play an important role in advocating for patients, implementing rehabilitation programmes, and coordinating with/working alongside other health professionals (Ford et al., 2022). The role occupational therapists have in contributing to the health and welfare of people in custody warrants synthesis.

The aim of this scoping review was to synthesise the literature regarding the role of occupational therapists in custodial settings, the challenges they face, and the impact of occupational therapy on detainee outcomes. The research questions underpinning this review were as follows: (i) What is the role of occupational therapists working in custodial settings? (ii) What are the challenges experienced by occupational therapists working in custodial settings? And (iii) what are the impacts of occupational therapy on outcomes for custodial detainees?

## 2 METHODS

### 2.1 Study design

A systematic scoping review of the literature was undertaken guided by the Joanna Briggs Institute (JBI) approach (Peters et al., 2020) and reported using the Preferred Reporting Items of Systematic reviews and Meta‐Analysis for Scoping Reviews (PRISMA‐ScR) Guidelines (Tricco et al., 2018). Within this framework, we considered the population to be occupational therapist(s); the context to be custodial settings (jail/prison, short‐ or long‐term detainment, and forensic facilities); and the concept to be the role of, challenges to, and the outcome/s of occupational therapy.

### 2.2 Positionality statement

The first (EE) and senior (JC) authors are registered nurses holding conjoint academic and health service positions, with a shared interest in strengthening the health‐care workforce and improving care for vulnerable populations. The second author (SW) is a registered and practicing occupational therapist. All three authors are experienced in conducting literature reviews with (EE) and (JC) having previously published multiple scoping reviews in peer‐reviewed journals.

### 2.3 Data collection

An iterative approach was used to develop the search strategy. An initial search of CINAHL was conducted to test key terms and search strings. The terms and search strings were then revised and a comprehensive search of CINAHL, EMBASE; and the Criminal Justice Database (PROQUEST) was undertaken. The search strategy included different combinations of Medical Subject Headings (MeSH) terms and keywords relevant to occupational therapy (‘occupational therap*’ OR OT OR OTA) and custodial settings (custodial* OR Prison* OR Incarcerat* OR Jail* OR ‘Correction* Facilities’ OR ‘Forensic psychiatric hospital’ OR detention OR ‘Watch house’ OR Custody* OR gaol* OR penitentiary). Search terms were combined with either OR and AND to create search strings. Stemming, using an ‘*’ and wildcard ‘?’, was also part of the search process. The search terms and strategy were developed with the support of an expert Health Librarian. Table S1 presents the electronic search strategy for the CINAHL database.

### 2.4 Inclusion and exclusion criteria

Inclusion and exclusion criteria for this literature review are displayed in Table 1. Included were studies published between January 2013 and May 2024 in English, full‐text, peer‐reviewed original research, pertaining to occupational therapists/therapy in custodial settings. Title and abstract screening were performed by two reviewers (EE and JC), with moderation performed by a third reviewer (SW). Full‐text studies were screened for inclusion by two reviewers (EE and JC) with disagreement resolved with a third reviewer (SW).

**TABLE 1:** Inclusion and exclusion criteria.

Inclusion criteria	Exclusion criteria
Academic journals Peer‐reviewed journal articles Original research papers 1 January 2013 to 1 May 2024 Published in English Scope of occupational therapy only in custodial/forensic settings Community re‐entry Occupational therapy programmes implemented in custodial settings Occupational therapy roles within allied health services Occupational therapy in custodial mental health settings	Non‐English Literature reviews Animal studies Occupational therapy in non‐forensic settings Interventions for custodial setting staff Effects of incarceration without occupational therapy intervention Drug interventions Refugee detention settings or immigration removal centres Conference presentations Abstracts only Thesis—Published PhDs Editorials Policy papers White papers Factors influencing an occupational therapist to work/not work in custodial settings Occupational therapist training services Other non‐occupational therapy health interventions General challenges faced by inmates No evidence of ethical approval or waiver

### 2.5 Charting and appraising the data

Based on the JBI approach (Peters et al., 2020), a data extraction table was developed by members of the authorial team. Information extracted from each paper was entered into a Microsoft Word 2018 table (Microsoft Corporation; Redmond, Washington USA). This information included: author(s), year of publication, country of study, research questions/aims, setting and sample, role of occupational therapist, other key findings. The Mixed Methods Appraisal Tool (MMAT) (Hong et al., 2019) was used to conduct a critical appraisal of included studies. In the first instance, the publications were assessed for (i) clear research questions or aims and (ii) how data were collected, in relation to the question/aim. The remaining MMAT considerations were then applied to the included publications according to the study design (Hong et al., 2019).

### 2.6 Synthesis of results

Data extracted from the included studies and presented in the tables were synthesised. Based on study characteristics and inclusion criteria, a descriptive‐analytical framework was used (Adams et al., 2023).

## 3 RESULTS

**FIGURE 1:**
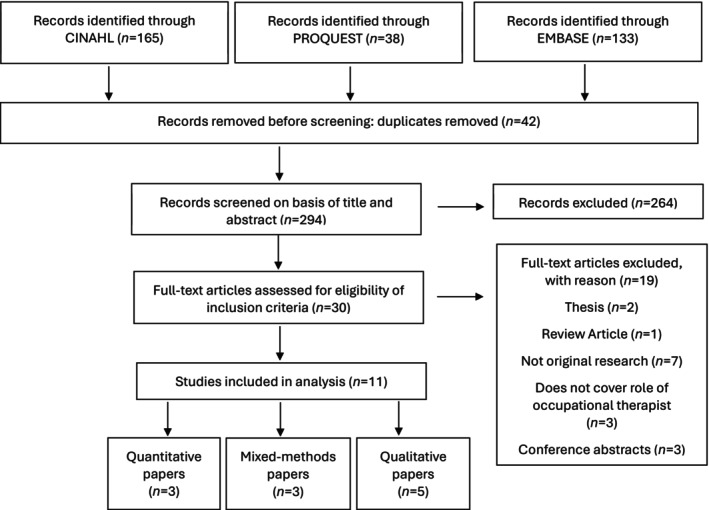
Modified PRISMA flow diagram (Liberati et al., 2009).

In total, 11 papers met the criteria for inclusion (see Figure 1). A summary of articles included in this review is displayed in Table 2.

**TABLE 2:** Summary of included articles.

Author/s (year) Country	Research Question(s)/aims	Design, tool/s, setting, and sample	Intervention/s	Role of the occupational therapist	Key findings	Limitations
Crabtree, Ohm, et al. (2016) USA	To explore the strength and weaknesses of an informal education programme and identify elements of the programme valued by participants	**Design:** Participatory Action Research (PAR) **Setting:** Indianapolis Re‐Entry Education Facility **Tool/s:** Semi‐structured interviews ranging 30–45 min **Sample:** *N* = 27 *n* = 3 resident investigators *n* = 24 former participants	Small group and individual consultation for men who have been incarcerated for ≥10 years, based on: technology socialisation finances employment health and education ***Frequency:*** Alternating topics held once weekly from 8:00 AM–3:30 PM. Participants attended a total of five full day sessions.	To design and facilitate an individualised intervention with inmates. The sessions were co‐facilitated with occupational therapy students.	**Five key themes emerged from the interviews:** Doing (role plays and simulations) Information (variety of information shared and the manner in which it was shared [i.e., brochures and PowerPoints]) Re‐entry fears (reconnecting with society post incarceration) Technology (current disconnect with technological advances due to incarceration and the impact on technology knowledge can have on successful re‐entry) Self‐worth (personal perceptions of self‐worth of the prisoners)	The qualitative nature makes it difficult to generalise findings. As the researchers were a part of the study, some researcher bias may exist.
Crabtree, Wall, and Ohm (2016) USA	(1) To critically reflect on the challenges faced throughout the evaluation process, and the environmental barriers and benefits experienced during conducting the OTCLS programme and an informal occupational therapy education programme (2) Reflect on power differentials existing in prison settings	**Design:** Participatory Action Research (PAR) **Setting:** Indianapolis Re‐Entry Education Facility **Sample:** Current and former inmates that have participated in the programme were involved in a semi‐structured interview used to evaluate the programme	Small group and individual consultation for men who have been incarcerated for ≥10 years, based on: technology socialisation finances employment health and education ***Frequency:*** Alternating topics held once weekly	To design and facilitate an individualised intervention with inmates. To decrease negative effects of occupational deprivation in prison settings by introducing engagement in meaningful tasks The sessions were co‐facilitated with occupational therapy students.	The use of PAR allowed all collaborators to engage as co‐occupation and gain a more in‐depth understanding of occupational justice. Occupational therapists and other health professionals must maintain healthy relationships with internal staff in prison settings, be flexible with the schedules of people that are detained, and follow the rules and regulations to be in a position of potentially advocating for enhancement in occupational justice Despite diverse backgrounds, personal growth and an appreciation for each other's humanity was achieved through co‐occupation.	Challenging to involve participants with diverse backgrounds in the interview due to existing rules and regulations
Dowdy et al. (2020) USA	(1) What are the correlations between youths' histories of toxic stress, their sensory processing patterns, and AoV committed within the juvenile correctional facility? (2) Is there a difference between youths' rates of violent behaviour before and after sensory‐based occupational therapy interventions? (3) How do incarcerated youth who received sensory‐based occupational therapy interventions describe their experiences with this intervention?	***Design:*** Mixed methods design (qualitative and quantitative) using: correlational design quasi‐experimental design qualitative (interviews) design ***Tool/s:*** ACE survey tool A/ASP survey tool author‐developed interview guide ***Setting:*** Maximum security youth juvenile correctional facility. ***Sample:*** Quantitative *n* = 76 incarcerated male records aged between 15 and 20 years Qualitative *n* = 76 currently incarcerated men 18+ years old who have undergone a min of 300 min of occupational therapy interventions	**Group based activities and sessions focussing on:** learning and practice using adaptive sensory‐based regulation strategies improving life skills and other functional activities **Possible activities included:** finger painting with shaving cream creating rain sticks drawing listening to and creating rhythmic music exploring aromatherapy or fidgets practicing meditation playing kinetic sand games making peel‐and‐stick sand art **Duration and frequency:** no standardisation treatment duration sessions held 2–4 times a month as deemed clinically appropriate	To conduct occupational therapy sessions with individuals currently incarcerated in a maximum‐security prison. Occupational therapy sessions include activities improving emotional regulation, sensory regulation, and life skills such as employability and communication.	**Quantitative:** AoV significantly decreased with OT support (from *M* = 037 to *M* = 0.020; *t* [75] ¼ 5.25, *p* ¼ < .001) Decreased violence after undergoing sensory/emotional regulation activities **Qualitative:** **Key themes (Post‐Occupational Therapy):** ***Theme 1:*** Youth recognising and understanding emotional regulation ***Theme 2:*** Perceptions of occupational support ***Theme 3:*** Empowerment and optimism for the future	Single site study Data utilised in chart review was limited to that already collected at the facility for the purposes of clinical evaluation and treatment. ACE and A/ASP validity may be influenced by the context and setting as well from court documents and by self‐report.
Dowdy et al. (2023) USA	To investigate changes in emotional state identified by incarcerated youth after receiving sensory‐based occupational therapy treatment	***Design:*** A quasi‐experimental retrospective design ***Tool/s:*** DSM‐IV for mental health diagnoses ACE Survey Emotions Intervention Tool ***Setting:*** *N* = 2 Midwestern juvenile correctional facilities ***Sampl**e:* Incarcerated youth ages 12–20 years *(total participant numbers not reported)*	Sensory‐based interventions Meaningful activity interventions Mixed interventions	To facilitate activity as chosen by the patient.	**Intervention participation** 32% sensory‐based intervention 25% meaningful activity 43% mixed intervention **Most chosen activities** ***Sensory‐based intervention:*** music, fidgets, putty, slime, or talking ***Meaningful activity intervention:*** crafts, discussing zones of regulation, playing games to better understand the zones of regulation, or playing a meme emotion game to increase awareness of emotions. ***Mixed intervention:*** music or scented lotion paired with talking, stress ball or fidget making, a focus game, or a craft Average emotional scores improved post intervention (3.01 [SD = 2.50] vs. 1.45 [SD = 1.62]) ***Pre* vs *post intervention emotion word stated:*** calm *n* = 78 vs *n* = 132, happy n = 41 vs. *n* = 69, chill *n* = 33 vs *n* = 45, tired *n* = 23 vs. *n* = 17 ***Impact of occupational therapy (n = 10) ‘a lot’ responses*** *n* = 5 Use skills learned *n* = 5 Occupational therapy has helped change behaviour *n* = 4 Occupational therapy has helped control emotion *n* = 6 Occupational therapy has helped name and recognise emotions ***Emotion identification (N = 11) ‘okay’ and ‘really good/great’ responses*** Ability to name your emotions *n* = 10 Ability to control emotions *n* = 8 Themes from the open‐ended questions included: coping strategies life skills how to talk about their emotions before acting	Survey data was limited to participants who were at least 18 years old due privacy and consent issues. Single site study precludes the ability to generalise the findings to other correctional facilities.
Gonzalez et al. (2023) USA	To determine feasibility and efficacy of an occupational therapy programme for community re‐entry.	***Design:*** Mixed methods ***Tool/s:*** ***Quantitative*** demographic survey PROMIS assessments VISPDAT Activity Card Sort‐Advancing Inclusive Assessment ***Qualitative:*** Detainee exit interview Behavioural health interview Staff interviews ***Setting:*** Midwestern Department of Corrections (DOC) Community Supervision Centre ***Sample:*** *N* = 5	13 occupational therapist led group sessions: one introductory group to gauge interest in recreational activity, six pickleball groups at the local recreational centre, one exercise group in the CSC, two leisure/holiday bingo activities in the CSC, one structured Stages of Change group, one check in via telehealth during COVID‐19, and one guest speaker lecture. On average, there was seven members in each group ranging from four to 11 participants.	To facilitate group sessions	***Demographic:*** *N* = 5 males Mean age 34.4 (ranging from 21.2–44.8) *n* = 4 Caucasian *n* = 1 African American ***Client‐centred goals developed at the start of care included:*** continuation of sobriety meeting set goals completing job applications finding housing increasing leisure participation identifying, and using coping strategies to manage emotions budgeting exploring schooling options reducing frequency of smoking medication management ***Goal outcomes:*** 54.8% were met 29% were partially met (i.e., had made some progress) 9.7% were unmet 6.5% were requested to be put on hold ***Significant health changes were reported (1 > SD):*** self‐efficacy managing emotions anxiety sleep disturbances Moderate changes were seen in reduced feelings of social isolation (0.5 > 1SD). ***Themes generated from detainee exit interviews:*** benefits of getting out of the CSC, accountability, and structure, gaining life skills, client‐centeredness. ***Themes generated from staff perceptions of program feasibility and efficacy interviews:*** Initial hesitancy transformed into increased engagement and behaviour Benefits of separation of roles Clients lacking life skills Asset to the probation and parole team	Due to the small sample size, the quantitative analysis is subject to fallibility, and alpha scores were not able to be determined from the PROMIS scores. COVID‐19 restrictions precluded the occupational therapists from completing the final few sessions as planned. The lack of blinding randomization and use of control groups limits the generalisability of the findings.
Jaegers et al. (2020) USA	To examine the implementation fidelity of an occupational therapy‐administered interprofessional re‐entry programme initiated in an urban jail	***Design:*** Retrospective, mixed quantitative, and qualitative design ***Setting:*** Midwestern urban jail and post‐release facility in the local St. Louis community. ***Sample:*** *N* = 63 Fully completed *n* = 24 Partially completed *n* = 33 Other *n* = 6	8‐week pre‐release programme. Participants develop individualised vision plan to set goals for education, employment, social networking, and leisure. Educational modules conducted daily, which addresses individualised needs of the participants. Module topics included: job seeking (e.g., resume writing, interview skills, skill assessment, and career exploration) literacy (e.g., health, financial, reading, and math, occupational) leisure skills communication emotional regulation health trauma healthy relationships community resources transition planning **Frequency:** 4‐days per week **Duration:** Education workshops were held for 2.5 per day	To administer an interprofessional re‐entry programme to incarcerated offenders. Referral of programme users to community occupational therapy (bridging community reintegration)	Implementation of pre‐release jail‐based services because of jail operations and community partnerships (facilitators) and overcoming institutional policies and environmental limitations (barriers) is feasible. Barriers and facilitators to programme implementation: social–ecological individual interpersonal institutional procedural university All who completed the full pre‐release programme and transitioned to the community (*n* = 15) initiated post‐release occupational therapy services.	Study conducted in urban setting and therefore may not be generalisable to regional or rural settings. Bias of interpretation of barriers and facilitators may have occurred due to the interaction between the researchers.
Muñoz et al. (2016) USA	The research team identified three objectives for the study: (1) Establish a baseline inventory of the occupational therapy educational training, intervention programming, and research occurring in criminal justice settings within the USA; (2) To describe the scope of practice, including identification of primary practice models guiding interventions, frequently used assessments and common individual and group interventions; and (3) To explore the feasibility of creating a network for occupational therapists working within criminal justice settings	**Design:** Descriptive retrospective survey design comprising of both quantitative and qualitative responses. ***Tool/s:*** Author‐developed scale **Sample:** *N* = 48 occupational therapists ***Setting:*** U.S. Criminal Justice Settings.	N/A	To predominantly work with incarcerated people by providing individual and group support in developing skills that foster independence and success post‐release.	**Educational training, intervention programming and research:** Majority had more than ≥6 years experience 69% possess post graduate or PhD qualifications 40% created service learning 57% have either conducted or supervised research within the correctional setting 51% work in traditional correctional settings 49% work in community settings **Scope practice/practice models:** 37.5% do not routinely use a practice model Majority (60.4%) do not routinely use non‐OT models to inform practice *Models reportedly used:* MOHO (29%) PEOP & PEO Model (20%) Cognitive Disability model (14.5%) Canadian Model of Occupational Performance and Engagement (12.7%) Occupational Adaption Model (12.7%) *Frequently used assessments:* Occupational Self‐Assessment Assessment of Motor and Process Skills Assessment of Communication and Interaction Skills Occupational Questionnaire Occupational Therapy Task Observation Scale Allen Cognitive Level Screen *Most used routine assessments:* Interview tools—COPM 51% Performance tools—ACL 54% Self‐reported tools—Sensory Profile 28% Observational tools—COTE Scale 40% Group interventions commonly addressed by ≥50% addressed: coping (67%) stress management (67%) goal setting (62%) employment (52%) leisure (52%) wellness skills (52%) Most frequently defined outcomes of interventions: development of community living skills (21.6%) work/employment skills (21.6%) interpersonal communication/social skills (23.3%) **Feasibility of creating a network:** 83% interested in joining special interest section for criminal justice occupational therapists. 79% supported the development of a position paper Web based approaches were preferred for networking	Recruitment was limited to the researchers personal database Survey used had not been previously validated.
Mynard et al. (2024) Australia	To explore and describe the work, context and professional reasoning of occupational therapists working in solitary confinement settings within a large forensic mental health service	***Design:*** Qualitative using semi‐structured interviews ***Setting:*** Forensic mental health service ***Sample:*** *N* = 11 occupational therapists	**Activity based interventions include the following:** Orientation Sensory modulation Physical activities Pen and paper activities Games Art and craft Food preparation	To facilitate activities with detainees held in solitary confinement.	Themes generated about the context and description of work of the occupational therapist: ***It's all about risk*** The physical environment is designed to manage risk Different philosophical approaches to managing risk Occupational therapy contribution to assessment and management of risk ***The work we do*** Indirect work advocating for patients Direct work with patients ***Why we do what we do*** Implementing theoretical concepts Belief in the power of occupation	The findings are limited to the thoughts and experiences of a purposive sample of occupational therapists from single site and therefore may not be generalisable to the wider occupational therapist community.
Shea and Siu (2016) USA	To explore the extent of engagement of male and female inmates aged 14 to 18 years old in structured play activities on topics such as interpersonal relationships, self‐awareness, cultural celebrations, and the transition to community	***Design:*** Exploratory retrospective study ***Tools:*** Artwork EOAQ AGAS ***Setting:*** Juvenile Justice Centre ***Sample:*** Incarcerated male and female youth between the ages of 14–18 (*N* not provided).	**Weekly 1‐h sessions for 48 weeks**. **Developmentally appropriate play occupations to help foster resilience:** Crafts Games with rules Interactive activities **Life skills addressed during sessions:** Pre‐vocational exploration Assertive communication Self‐management How to access community resources **Topics included in structured play:** Interpersonal relationships Self‐awareness Cultural celebrations Transition to community	To facilitate and group lead OTPP sessions.	***AGAS results*** **76–100% work completed:** Females 92% Males 83% **76–100% work relevant to content:** Females 97% Males 95% ***EOAQ results:*** **Engagement scores for females compared with male detainees (Mean, SD):** Interpersonal relationships (3.72, 0.15 vs. 4.49, 0.23) Self‐awareness (3.68, 0.12 vs. 4.16, 0.13) Cultural celebrations (4.21, 0.17 vs. 4.21, 0.19) Transition to community (4.46, 0.24 vs. 3.95, 0.12) **Top 5 EOAQ mean scores (M, SD):** Reflect the kind of person I am (4.11, 1.16) Express my creativity (4.10, 1.08) Help me express my personal values (4.08, 1.16) Help me achieve a sense of accomplishment (4.05, 1.09) Give me a sense of satisfaction (4.05, 1.15) **Themes relating to meaningfulness of activities generated from detainee responses:** Self‐awareness Self‐identity (learning about and being able to express oneself) Inspiring others Transition to community Financial literacy Cultural celebrations Expressing self‐realisation Gratitude Interpersonal relationships Relationship help Identifying desired traits and qualities in friends	Study conducted in single site and therefore may not be generalisable to other settings. The exploratory design, inhibited a systematic examination of the effectiveness of the OTTP interventions to be undertaken. The actual number of participants is unknown, impacting the reliability and generalisability of the findings.
Tan et al. (2015) Singapore	To evaluate the occupational therapy‐based rehabilitation programmes to provide therapy for male offenders within forensic settings, targeting the occupation of social interaction	***Design:*** Critical reflection on practice ***Setting:*** Psychiatric Housing Facility within a custodial prison setting ***Tool:*** Task Behavioural Scale (based on the Occupational Therapy Task Observation Scale & Comprehensive Occupational Therapy Evaluation) ***Sample:*** Male prisoners *N* = 50 MDT: P.T. psychiatrist *N* = 1 OT *N* = 2 Nurses *N* = 6	**Three‐phase programme and frequency:** ***Assessment Phase:*** occupational therapists build rapport with the participants. Two group sessions over 3 months ***Basic Phase:*** Prisoners learn to efficiently develop and maintain social relationships and reduce maladaptive coping behaviours using different occupations. Three sessions per week over 3 months ***Maintenance Phase***: Prisoners learn to develop life management skills: budgeting and employability. Three sessions per week over 6 months **Three‐tier GWP aimed to improve employability of prisoner**. ***Level One—Prisoners requiring close supervision:*** could engage in cleaning tasks. ***Level Two—Prisoners requiring some supervision:*** could engage in the role of serving food within the prison. ***Level Three—Prisoners requiring minimal supervision:*** could engage in a variety of worker roles such as cleaning, helping during medication rounds, serving food.	To establish and conduct an MDT programme that was aimed at creating meaningful occupations within a restrictive environment while managing offenders with mixed psychiatric conditions.	**Quantitative results from the TBS**: After 1‐year offenders showed an improvement in all functional domains of the adapted TBS. Improvements the TBS scores were sustained across all three phases. **Critical reflection:** ***Occupational therapists views:*** It is challenging to provide authentic occupations for offenders within the custodial prison setting ***Offenders view:*** Offenders appreciated the opportunity to choose topics in an environment where choice is often limited Group activities facilitated self‐reflection among programme offenders Activities such as colouring produced calming effects among some offenders Games played helped offenders to learn how to abide by rules	The critical reflection design precluded the ability to assume causation between occupational therapy intervention and reduction in offences committed.
Whiteford et al. (2020) Australia	The aim of the practice‐based enquiry project described in this article was, overall, to improve quality of life and the potential community reintegration of patients in a forensic hospital.	***Design:*** Practice‐based enquiry approach ***Setting:*** Forensic hospital ***Tool/s:*** Reflective practice Written forms Oral recordings ***Sample:*** Occupational therapists *N* = 9 commenced *n* = 5 completed	Monthly community of practice meetings approx. 2 h in duration	To participate in community of practice meetings and conduct analysis of the reflective accounts	Through the PBE process: participants' practice became more occupation‐centred, based, and focussed there was creation of more opportunities for patients occupational deprivation 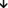 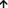 potential for community reintegration 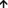 professional satisfaction and identity 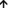 institutional ‘valuing’ of the occupational therapy service	Study conducted in single site and therefore may not be generalisable to regional or rural settings. Experience and availability of facilitator may have impacted processes and findings.

Abbreviations: ACE, Adverse Childhood Experiences; ACL Screen, Allen Cognitive Level Screen; AGAS, Analysis of Group Activity Scale, AoV = acts of violence; A/ASP, Adolescent/Adult Sensory Profile; CSC, Community Supervision Centre; COPM, Canadian Occupational Performance Measure; COTE Scale, Comprehensive Occupational Therapy Evaluation Scale; EOAQ, Engagement in OTTP (Occupational Therapy Training Program) Activities Questionnaire; GWP, General Workers Programme; IREF, Indianapolis Re‐Entry Education Facility; LOE, Level of Evidence; MOHO Screening Tool; *N*, total number; *n*, sub‐group number; N/A, not applicable; OCAIRS, Occupational Circumstances Assessment Interview and Rating Scale; OTTP, Occupational Therapy Training Program; OTCLS, Occupational Therapy Community Living Skills; PAR, Participatory Action Research; PBE, Practice Base Enquiry; P.T., part‐time; PROMIS, Patient‐Reported Outcomes Measurement Information System; PTSD, Post‐traumatic stress disorder; TBS, Task Behavioural Scale; USA, United States of America; VIA‐IS‐P, Values in Action Inventory of Strengths; VISPDAT, Vulnerability Index‐Service Prioritization Decision Assistance Tool; Vs, versus; 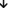, decreased; ≥, greater than; 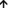, increase/d; ≤, less than; %, percentage; +, plus.

Of the 11 articles that met the inclusion criteria, eight were from the USA, two were from Australia and one was from Singapore. Study design varied with three of the included studies using a mixed methodology (Dowdy et al., 2020; Jaegers et al., 2020; Muñoz et al., 2016) and five were qualitative in nature (Crabtree, Ohm, et al., 2016; Crabtree, Wall, & Ohm, 2016; Mynard et al., 2024; Tan et al., 2015; Whiteford et al., 2020). The settings varied with three studies being conducted in juvenile correctional facilities (Dowdy et al., 2020, 2023; Shea & Siu, 2016), one adult maximum security and four being described as either forensic mental health (Mynard et al., 2024; Whiteford et al., 2020) or psychiatric housing facility within a prison setting (Tan et al., 2015). The remaining settings included prison re‐entry or community supervision facilities (Crabtree, Ohm, et al., 2016; Crabtree, Wall, & Ohm, 2016; Gonzalez et al., 2023; Jaegers et al., 2020; Muñoz et al., 2016).

### 3.1 Role of the Occupational Therapist

In all of the included studies, the occupational therapist's role involved direct detainee care (Crabtree, Ohm, et al., 2016; Crabtree, Wall, & Ohm, 2016; Dowdy et al., 2020, 2023; Gonzalez et al., 2023; Jaegers et al., 2020; Muñoz et al., 2016; Mynard et al., 2024; Shea & Siu, 2016; Tan et al., 2015; Whiteford et al., 2020). Skill development among detainees, such as communication, emotional and sensory regulation, fostering independence and enhancing employability post incarceration was the foci much of the occupational therapist roles described (Crabtree, Ohm, et al., 2016; Crabtree, Wall, & Ohm, 2016; Dowdy et al., 2020, 2023; Gonzalez et al., 2023; Jaegers et al., 2020; Muñoz et al., 2016; Shea & Siu, 2016; Tan et al., 2015). One publication described the occupational therapist role to involve onward referral of detainees to community‐based occupational therapy services (Jaegers et al., 2020), and another two of the included studies indicated that the occupational therapists worked within a multi‐disciplinary team as part of their role (Jaegers et al., 2020; Tan et al., 2015). One study reported the role to include occupational therapy student supervision and intervention facilitation (Crabtree, Wall, & Ohm, 2016).

Whereas most of the articles highlighted how occupational therapists complimented the broader health and custodial teams, within some of the articles, it was suggested that some occupational therapists were not necessarily working to their usual full scope of practice: *‘I don't think OTs [occupational therapists] … are allowed to do as much as they should, with things like clinical risk assessments … … think is definitely a gap or a downfall because I think OTs have a unique perspective in risk assessment’* (Mynard et al., 2024). The duality of the occupational therapist role in custodial settings (health‐care provider versus member of the custodial team) was also noted (Tan et al., 2015). Although a member of the custodial workforce, the role of the occupational therapist was viewed distinct and positively from other custodial staff by detainees, for example, ‘*you are out for the best interest of…us…you know and the CSC [Community Supervision Centre], it ain't so much…they treat us like criminals …*’ (Gonzalez et al., 2023) and *‘… just being able to go once a week and talk to somebody who I know actually cares and wants to help me do better …’* (Dowdy et al., 2020).

Both individual and group‐based interventions for detainees were described. In some instances, either one‐on‐one (Dowdy et al., 2020) or group‐based interventions (Jaegers et al., 2020) were discussed; however, two articles indicated interventions that comprised of both one‐on‐one and group‐based sessions (Crabtree, Wall, & Ohm, 2016; Tan et al., 2015). Frequency and duration of the interventions varied. The longest intervention was conducted over a 12‐month period (Tan et al., 2015). The frequency of the sessions ranged from several times per week (Jaegers et al., 2020) to a few times per month (Dowdy et al., 2020; Tan et al., 2015). One included article did not discuss an intervention (Muñoz et al., 2016). Improving employability either within the custodial setting or post‐release was the foci of many of the interventions described (Crabtree, Wall, & Ohm, 2016; Jaegers et al., 2020; Tan et al., 2015). In all the studies, the occupational therapist played a key role in leading or delivering the intervention.

### 3.2 Challenges experienced by occupational therapists working in custodial settings

Seven studies discussed the challenges experienced by occupational therapists in custodial settings (Crabtree, Wall, & Ohm, 2016; Gonzalez et al., 2023; Jaegers et al., 2020; Mynard et al., 2024; Shea & Siu, 2016; Tan et al., 2015; Whiteford et al., 2020). These challenges were classified into four broad categories: (i) engagement, (ii) environmental, (iii) governance, and (iv) resourcing.

#### 3.2.1 Engagement

When occupational therapists were first introduced to the custodial setting, both staff and detainees had limited understanding of the occupational therapist's role and position with the custodial setting, leading to initial hesitancy from both correctional staff and detainees in wanting to participate in a pilot intervention (Gonzalez et al., 2023). However, as described in other research, over time institutions became more appreciative of and willing to engage with occupational therapy once programmes had become more established (Whiteford et al., 2020).

Untreated mental health conditions, challenging group dynamics, and lack of family support were noted to negatively influence engagement with occupational therapy services offered in custodial settings (Jaegers et al., 2020). Not all detainees were able to complete occupational therapy programmes due to the length of detention (Jaegers et al., 2020), with engagement tending to be higher among women when compared with male detainees (Shea & Siu, 2016). Therapeutic interprofessional relationships between custodial staff and occupational therapists was important to improve detainee outcomes (Crabtree, Wall, & Ohm, 2016; Mynard et al., 2024; Tan et al., 2015).

#### 3.2.2 Environmental challenges

Restrictive environments, including physical barriers (Crabtree, Wall, & Ohm, 2016; Mynard et al., 2024), perceived risks around activities (for example cooking and outings) (Tan et al., 2015) as well as limited access to technology provides unique challenges to occupational therapists in custodial settings. Interventions that align with real‐life and employment skills are often hindered by intuitional constraints and were discussed by occupational therapists, detainees, or both groups (Crabtree, Wall, & Ohm, 2016; Tan et al., 2015). Low‐risk detainees were able to attend sessions face‐to‐face, whereas high‐risk detainees received occupational therapy through a safety barrier, such a large glass window, small opening or a letter‐box sized slot (Mynard et al., 2024). Custodial staff and the occupational therapists sometimes differed on their perception and understanding of ‘risk’ (Mynard et al., 2024). From an occupational therapy perspective, the custodial setting made providing authentic occupations for offenders challenging (Tan et al., 2015).

#### 3.2.3 Governance

Policy and legislation along with varying durations of detainment were challenging for the occupational therapist to navigate (Jaegers et al., 2020). Policies often regulated the types of activities, equipment permitted to be used or available in the rooms, and who was deemed safe or appropriate to participate in occupational therapy sessions. For example, detainees who were prescribed psychiatric medications were prevented from participating due to being deemed high‐risk (Tan et al., 2015), whereas others reported that use of technology was prohibited (Crabtree, Ohm, et al., 2016). Some detainees were noted to display deceptive behaviours intentionally breaching policies in order to take items back to their rooms (Mynard et al., 2024; Tan et al., 2015). From both the therapists and detainees' perspectives some policies made it challenging to implement authentic occupational therapy programmes (Crabtree, Ohm, et al., 2016; Mynard et al., 2024). Other governance and policy challenges noted were temporal and directly related to the COVID‐19 pandemic (Gonzalez et al., 2023).

#### 3.2.4 Resources

Most of the resourcing was discussed in relation to access to rooms, furniture and equipment and aligned with governance and environmental challenges. Two articles referred to human resourcing and funding. The limited availability of occupational therapy staff (3 days/week) was noted by custodial staff as a barrier in implementing occupational therapy in custodial settings (Gonzalez et al., 2023), whereas the success of another programme was accredited to the financial support to fund a full‐time occupational therapist (Jaegers et al., 2020).

### 3.3 Impacts of occupational therapy on outcomes for detainees

For detainees who engaged with occupational therapy programmes while in custody, aggression and recidivism rates decreased (Crabtree, Wall, & Ohm, 2016; Dowdy et al., 2020; Jaegers et al., 2020; Tan et al., 2015). Emotional regulation also improved, with acts of violence (Dowdy et al., 2020) and self‐reported feelings of social isolation both decreasing among detainees participating in occupational therapy programmes (Gonzalez et al., 2023). Furthermore, detainees who completed occupational therapy programmes while in custody were more likely to engage with post‐release occupational therapy services (Jaegers et al., 2020).

The benefits of occupational therapy within custodial settings were recognised by detainees in a number of studies (Dowdy et al., 2023; Gonzalez et al., 2023). Participants stated that they enjoyed the occupational therapy sessions (Dowdy et al., 2020; Gonzalez et al., 2023) and as a result of occupational therapy now had a better understanding of their emotions (Dowdy et al., 2020, 2023; Gonzalez et al., 2023). One programme recruited an additional occupational therapist a year after it begun due to the perceived programme effectiveness and improved outcomes (such as improved relationships with others, ability and willingness to show initiative, and developing a sense of pride in their work) among detainees with plans to expand the service to other institutions (Tan et al., 2015).

### 3.4 Quality appraisal

Table 3 presents the results of the quality appraisal for each of the 11 included studies. The MMAT appraisal indicated that all included studies met the initial MMAT screening questions, and after further appraisal, three of the 11 studies scored ‘yes’ for all appraisal items.

**TABLE 3:** Appraisal of included studies using the Mixed Methods Appraisal Tool (Hong et al., 2019).

Qualitative	Is the qualitative approach appropriate to answer the research question?	Are the qualitative data collection methods adequate to address the research question?	Are the findings adequately derived from the data?	Is the interpretation of results sufficiently substantiated by data?	Is there coherence between qualitative data sources, collection, analysis, and interpretation?
Crabtree, Ohm, et al. (2016)	Yes	Yes	Yes	Yes	Yes
Crabtree, Wall, and Ohm (2016)	Yes	Yes	Cannot tell	Yes	Cannot tell
Mynard et al. (2024)	Yes	Yes	Yes	Yes	Yes
Tan et al. (2015)	Yes	Yes	Cannot tell	Cannot tell	Cannot tell
Whiteford et al. (2020)	Yes	Cannot tell	Cannot tell	Yes	Cannot tell
***Quantitative descriptive***	***Is the sampling strategy relevant to address the research question?***	***Is the sample representative of the target population?***	***Are the measurements appropriate?***	***Is the risk of nonresponse bias low?***	***Is the statistical analysis appropriate to answer the research question?***
Dowdy et al. (2020)	Cannot tell	Cannot tell	Yes	Cannot tell	Yes
Muñoz et al. (2016)	Cannot tell	Yes	Yes	Cannot tell	Yes
Shea and Siu (2016)	Cannot tell	Cannot tell	Cannot tell	Cannot tell	Yes
***Mixed methods***	***Is there an adequate rationale for using a mixed methods design to address the research question?***	***Are the different components of the study effectively integrated to answer the research question?***	***Are the outputs of the integration of qualitative and quantitative components adequately interpreted?***	***Are divergences and inconsistencies between quantitative and qualitative results adequately addressed?***	***Do the different components of the study adhere to the quality criteria of each tradition of the methods involved?***
Dowdy et al. (2023)	Yes	Yes	Cannot tell	Yes	Cannot tell
Gonzalez et al. (2023)	Yes	Yes	Yes	Yes	Yes
Jaegers et al. (2020)	Yes	Yes	Yes	Yes	Cannot tell

## 4 DISCUSSION

This scoping review systematically synthesised the evidence regarding the role of occupational therapists working in custodial settings. The findings of this review contribute to furthering the understanding of the occupational therapist's role within custodial settings. Specifically, key findings from this review include the role of the occupational therapists, challenges experienced by occupational therapists, and the impact on detainees.

### 4.1 Role

The World Federation of Occupational Therapists described the primary role of occupational therapists as ‘enabling people to participate in activities of everyday life’ (World Federation of Occupational Therapists, 2020). The role of occupational therapists also encompasses supporting individuals to engage in meaningful occupations where modification of the role or environment might be required, enhancing their abilities to engage in occupations they want, need, and must do (World Federation of Occupational Therapists, 2020). Within custodial settings, occupational therapists uniquely adapt this role focussing on the development and maintenance of life skills both during and after incarceration. Our findings indicate custodial‐based occupational therapists play a crucial role in conceptualising, implementing and leading interventions that address detainee mental health issues, substance misuse, low literacy levels, and social isolation. They employ a distinctive approach by facilitating engagement in meaningful activities, skill‐building programmes, and rehabilitation initiatives. Additionally, they support emotional regulation, coping mechanisms, and educational preparation, which are critical for successful reintegration into society.

### 4.2 Challenges

Working in custodial settings can present unique challenges for health staff (Jeker et al., 2023). Consistent with the wider custodial health literature, the duality of roles (Simon et al., 2020) and lack of available staffing (Dennard et al., 2021) were recognised as challenges within this review. The restrictive nature of incarceration settings further contributed to the challenges reported in the literature (Tan et al., 2015). Ways to overcome the challenges of working in custodial settings may include enhanced interdisciplinary collaboration, increased staffing to ensure balanced caseloads (Tadros et al., 2023), and targeted professional development aimed at preparing occupational therapists for the complexities of this environment (Dennard et al., 2021; Jeker et al., 2023). Implementing policies that clarify the therapeutic versus custodial responsibilities of health professionals may also help reduce role strain (Pont et al., 2012). Additionally, promoting a trauma‐informed care approach could improve the quality of interventions while supporting the psychological well‐being of both staff and detainees (Dennard et al., 2021).

Custodial health is exceptionally complex and is recognised as a clinical specialty requiring a specialised and unique clinical skill set (Simon et al., 2020). A lack of homogeneity among the detainee population (Borzycki, 2005) along with the numerous challenges and disadvantages experienced by detainees, contribute to the complexity of care delivery. Some of the notable challenges include age (very young or very old), gender (in particularly women with dependent children), and disability (Baldry et al., 2012; Haesen et al., 2019; Mir et al., 2015). Indigenous populations and people of colour are often over‐represented in custodial settings (U.S. Department of Health and Human Services, 2020; Queensland Health and Queensland Corrective Service, 2020). High rates of mental health problems, substance misuse and low literacy levels are evident among the detainee population (Creese, 2016; Mir et al., 2015; Young et al., 2018), and social isolation and chronic disease are common concerns (Queensland Health and Queensland Corrective Service, 2020).

The diversity of detainees (i.e., age, gender, and cultural background), the over‐representation of indigenous populations, complex health needs along with the varying lengths of incarceration, and/or individual security concerns highlight the need for multi‐faceted or multi‐modal approaches. Single or generic programmes are often not viable in addressing these challenges (Borzycki, 2005). Challenges with under‐staffing and concerns associated with governance and infrastructure, as highlighted in this review, would also make delivering multi‐modal or multi‐faceted programmes difficult. To mitigate this, additional resources, funding and workforce support is needed to maintain and expand opportunities for occupational therapy in custodial settings.

### 4.3 Impact

Few countries report the costs of health‐care services in prison and consistency in reporting is lacking (Sridhar et al., 2018). The annual overall prison operating costs and health‐care expenditure as a percentage of operating expenditure has been reported to vary from 4% in Ireland to 9% in the UK (Scotland) and Australia, and 18% for State Prisons in the USA (Sridhar et al., 2018). Investing in prison health (and therefore the health of prisoners) has benefits both during and post incarceration. Typically, custodial health has not been salutogenic and has more‐so situated itself in a biomedical paradigm focussing on chronic disease management, effects of substance misuse, mental health, and acute medical/surgical conditions (Woodall et al., 2022). However, this medically focussed approach may not comprehensively address the acute and chronic social determinants of health such as homelessness, low literacy, and lack of employability post incarceration (Australian Institute of Health and Welfare, 2022b). These factors along with the impact of chronic disease and acute conditions on prisoners' participation in activities of daily living, may contribute to the criminogenic cycle (Woodall et al., 2022). In contrast, occupational therapy care delivery is often informed by the biopsychosocial model (Gentry et al., 2021). By integrating the biological, psychological, and social factors into care delivery, custodial‐based occupational therapy recognises that a detainee's overall well‐being and capacity to engage in meaningful activities during and post‐incarceration is shaped by their mental health, environment and social roles. As highlighted in this review, occupational therapists working in custodial services are uniquely positioned to address acute and chronic social determinants of health, through targeted interventions during incarceration, ultimately supporting improved detainee outcomes post‐release.

Reconviction and re‐imprisonment rates are as high as 63% in some countries (Yukhnenko et al., 2019). Reducing recidivism through means of increasing employability, reducing aggression, and improving general literacy and numeracy skills, is often a key goal of interventional programmes within prisons (Piacentini et al., 2018; Piquero & Rocque, 2020; Vandala, 2019). As highlighted by this review, implementing interventions, or conducting programmes that focus on enhancing or improving inmate emotional regulation, life skills, and self‐management is central to the work of occupational therapists in custodial settings (Crabtree, Wall, & Ohm, 2016; Dowdy et al., 2020; Jaegers et al., 2020; Tan et al., 2015). Relative to this statement, it is essential to acknowledge that in the studies used in this scoping review, occupational therapists expressed that they found it challenging to carry out occupational therapy interventions due to the restrictive nature of the custodial environment. This is an area that can be explored in further research (Tan et al., 2015). Consistent with other interventional studies (Docherty et al., 2021; Zarling et al., 2021) the occupational therapist‐lead programmes included in this review also saw a reduction of recidivism rates and aggression among prisoners (Crabtree, Wall, & Ohm, 2016; Dowdy et al., 2020; Jaegers et al., 2020; Tan et al., 2015).

Whereas health economics and the long‐term financial impact was not the foci of the studies included in this review, based on the improved outcomes and reduced recidivism rates, custodial based occupational therapy may contribute to alleviating some of the financial burden of prison health expenditure. Further research exploring the health economics associated with occupational therapy in custodial settings is warranted if a comprehensive understanding of its impact is to be achieved.

### 4.4 Limitations of studies included in this review

The overall quality of studies included in this review was mixed, with only three of the 11 included studies meeting all of the MMAT criteria. This indicates that while some high‐quality evidence exists, much of the available evidence has methodological or reporting limitations, highlighting the need for more rigorous study designs and subsequent publications in the area. The majority of publications in this review (eight out of 11) originate from the USA, which may limit the capture of diverse models of care and the representation of occupational therapy practice in other jurisdictions such as the UK, Canada or Australia. Additionally, it is challenging to establish direct causation between the occupational therapy programmes/interventions, recidivism rates and the quality of the participants' lives. First, the studies included in this review lacked randomisation. This may have been because of ethical considerations. There may have also been other types of rehabilitation or therapy sessions being carried out in the setting at the same time or other potential confounding factors not reported on within the studies.

### 4.5 Recommendations for practice and research

This review has highlighted the adaptability of occupational therapy in restricted settings such as prisons, jails, forensic mental health or other custodial setting. However, it is clear from this review that current models of care for custodial‐based occupational therapists are inconsistent, with the use of specific frameworks often being ad hoc. There is limited clarity on how programmes were developed and if the unique needs of the detained population are being met. Furthermore, it is unclear from the evidence how many occupational therapists work in custodial settings or what an ideal ratio of occupational therapist to detainee might be. As previously highlighted, the majority of evidence included in this review originates from the USA and therefore not necessarily representative of occupational therapy undertaken outside of America. The evident paucity of international literature documenting the role, challenges and impact of occupational therapists in custodial settings calls for further research and prioritisation from other countries, if a comprehensive understanding is to be achieved. The dominance of literature from the USA, despite occupational therapy being well established in places such as Australia and the UK, may be suggestive that more occupational therapists undertake higher degree research and training in the USA compared with other countries.

Future research might include studies designed to (i) develop a clear understanding of the number of, educational qualifications and current scope of practice of occupational therapists currently working in custodial settings across varying jurisdictions; (ii) understand the needs of occupational therapists, detainees, and correctional services in different countries and contexts; and (iii) develop, test, and evaluate short and long‐term outcomes from models of care designed to overcome barriers within custodial settings that may exist while aiming to improve health outcomes for detainees.

Even though the current evidence is sparse, and more research relating to occupational therapy in custodial settings is needed, internationally the societal challenges that often underpin or contribute to increased likelihood of criminal behaviour are common (Butler et al., 2011; Kinner & Butler, 2017; Nishar et al., 2023; Young et al., 2018). Understanding this, and in the absence of locally developed evidence, occupational therapists may benefit from looking to other jurisdictions with similar cultures, judicial systems, custodial settings, and occupational therapy models when implementing or refining custodial‐based occupational therapy in their local context.

### 4.6 Limitations

This scoping review has some limitations. Only three databases were searched by one author (EE) with the support of a Health Librarian and co‐author (JC). Although, the search strategy was comprehensive, there is the potential for missed studies or data collection bias. Only studies published in English between 2013 and 2024 were included, precluding any non‐English publications and publications outside of these search dates that may have contributed to the understanding of the role of occupational therapists in custodial settings. Furthermore, we excluded grey literature from our search strategy. Thus, our review does not capture or discuss other potentially valuable aspects from report types such as policy documents and practice guidelines. To remedy this, we suggest broadening subsequent reviews to include policy and practice guidelines for occupational therapists working in custodial settings. Despite the limitations outlined here and in Section 4.4, this review does highlight the state of evidence and the gaps in understanding of the role of occupational therapists working in custodial settings and serves as a valuable starting point for future potential cross‐cultural studies. Furthermore, our review focussed solely on the role of occupational therapists working in correctional settings. We suggest conducting a more comprehensive review critiquing the role of occupational therapists in other less‐traditional non‐hospital settings to gain a broader understanding of the scope and settings in which occupational therapy is practiced internationally.

## 5 CONCLUSION

Despite the limited evidence available, the role of occupational therapists in custodial settings appears to be consistent with the World Federation of Occupational Therapists by enhancing the ability of people to engage in positive occupations to enable them to participate in activities they deem meaningful. In addition, the (often poor) social determinants of health of the detainee population, coupled with the restrictive nature of custody, mean that integrating occupational therapists into the custodial health‐care team can strengthen the team's overall effectiveness by adding a valuable perspective and skill‐set to comprehensively address functional, environmental and psychosocial factors essential to holistic detainee care. Specifically occupational therapists can play a key role in custodial care, by contributing to the development of life skills, reducing aggression and reducing recidivism rates, thereby improving long‐term detainee outcomes. Further research is required to ascertain the full scope and impact of occupational therapists working in custodial facilities.

## AUTHOR CONTRIBUTIONS

All authors meet the criteria for authorship. **Elizabeth Elder:** Conceptualisation; methodology; validation; data curation; formal analysis; investigation; writing—original draft; visualisation; writing—review and editing. **Shannon Werner:** Validation; data curation; formal analysis; writing—original draft; visualisation; writing—review and editing. **Julia Crilly:** Conceptualisation; methodology; validation; formal analysis; investigation; writing—original draft; visualisation; writing—review and editing. All authors approved the final version to be published and agreed to be accountable for all aspects of the work [Correction added on 10 February 2026, after first online publication: ‘Oam’ has been deleted from Julia Crilly's name.].

## CONFLICT OF INTEREST STATEMENT

The authors have no conflict of interest to declare.

## Supporting information


**Table S1.** Search strategy of CINAHL database.

## Data Availability

Data sharing is not applicable to this article as no new data were created or analyzed in this study.

## References

[aot70042-bib-0001] Adams, F. , Zimmerman, P.‐A. , Sparke, V. , & Mason, M. (2023). Towards a framework for a collaborative support model to assist infection prevention and control programmes in low‐and middle‐income countries: A scoping review. International Journal of Infection Control, 19. 10.3396/ijic.v19.21851

[aot70042-bib-0003] Australian Institute of Health and Welfare . (2020). Health of prisoners. Canberra Retrieved from https://www.aihw.gov.au/reports/australias-health/health-of-prisoners

[aot70042-bib-0004] Australian Institute of Health and Welfare . (2022a). Health of prisoners. Canberra. https://www.aihw.gov.au/reports/australias-health/health-of-prisoners

[aot70042-bib-0005] Australian Institute of Health and Welfare . (2022b). Social determinants of health https://www.aihw.gov.au/reports/australias-health/social-determinants-of-health

[aot70042-bib-0006] Baldry, E. , Dowse, L. , & Clarence, M. (2012). People with mental and cognitive disabilities: pathways into prison. Background Paper for Outlaws to Inclusion Conference

[aot70042-bib-0007] Baybutt, M. , & Chemlal, K. (2016). Health‐promoting prisons: Theory to practice. Global Health Promotion, 23(1_suppl), 66–74. 10.1177/1757975915614182 27199019

[aot70042-bib-0008] Borzycki, M. (2005). Interventions for prisoners returning to the community: A report prepared by the Australian Institute of Criminology for the Community Safety and Justice Branch of the Australian Government Attorney‐General's department. Australian Institute of Criminology.

[aot70042-bib-0009] Butler, T. , Indig, D. , Allnutt, S. , & Mamoon, H. (2011). Co‐occurring mental illness and substance use disorder among Australian prisoners. Drug and Alcohol Review, 30(2), 188–194. 10.1111/j.1465-3362.2010.00216.x 21355926

[aot70042-bib-0010] Chow, W. S. , & Priebe, S. (2016). How has the extent of institutional mental healthcare changed in Western Europe? Analysis of data since 1990. BMJ Open, 6(4), e010188. 10.1136/bmjopen-2015-010188 PMC4854016 27130161

[aot70042-bib-0011] Crabtree, J. L. , Ohm, D. , Wall, J. M. , & Ray, J. (2016). Evaluation of a Prison Occupational Therapy Informal Education Program: A Pilot Study. Occupational Therapy International, 23(4), 401–411. 10.1002/oti.1442 27774682

[aot70042-bib-0012] Crabtree, J. L. , Wall, J. M. , & Ohm, D. (2016). Critical reflections on participatory action research in a prison setting: Toward occupational justice. Occupation, Participation and Health, 36(4), 244–252. 10.1177/1539449216669132 27647111

[aot70042-bib-0013] Creese, B. (2016). An assessment of the English and maths skills levels of prisoners in England. London Review of Education, 14(3), 13–30. 10.18546/LRE.14.3.02

[aot70042-bib-0014] Dean, K. (2020). Literature Review: Forensic Mental Health Models of Care. Mental Health Commission of New South Wales.

[aot70042-bib-0015] Dennard, S. , Tracy, D. K. , Beeney, A. , Craster, L. , Bailey, F. , Baureek, A. , Barton, M. , Turrell, J. , Poynton, S. , & Navkarov, V. (2021). Working in a prison: Challenges, rewards, and the impact on mental health and well‐being. Journal of Forensic Practice, 23(2), 132–149. 10.1108/JFP-12-2020-0055

[aot70042-bib-0016] Di Tommaso, A. , Isbel, S. , Scarvell, J. , & Wicks, A. (2016). Occupational therapists' perceptions of occupation in practice: An exploratory study. Australian Occupational Therapy Journal, 63(3), 206–213. 10.1111/1440-1630.12289 27116946

[aot70042-bib-0017] Docherty, M. , Lieman, A. , & Gordon, B. L. (2021). Improvement in emotion regulation while detained predicts lower juvenile recidivism. Youth Violence and Juvenile Justice, 20(2), 164–183. 10.1177/15412040211053786

[aot70042-bib-0018] Dowdy, R. , Estes, J. , Linkugel, M. , & Dvornak, M. (2020). Trauma, sensory processing, and the impact of occupational therapy on youth behavior in juvenile corrections. Occupational Therapy in Mental Health, 36(4), 373–393. 10.1080/0164212X.2020.1823930

[aot70042-bib-0019] Dowdy, R. , Estes, J. , McCarthy, C. , Onders, J. , Onders, M. , & Suttner, A. (2023). The influence of occupational therapy on self‐regulation in juvenile offenders. Journal of Child & Adolescent Trauma, 16(2), 221–232. 10.1007/s40653-022-00493-y 36340267 PMC9628343

[aot70042-bib-0020] Edwards, K. (2021). Prisoners' Perspectives on Limited Rehabilitative Program Opportunities. Qualitative Report, 26(4), 1128–1149. 10.46743/2160-3715/2021.4495

[aot70042-bib-0021] Esposito, M. (2015). Women in prison: Unhealthy lives and denied well‐being between loneliness and seclusion. Crime, Law and Social Change, 63, 137–158. 10.1007/s10611-015-9561-y

[aot70042-bib-0022] Fair, H. , & Walmsley, R. (2024). World prison population list (14th ed.). Institute for Crime & Policy Research. https://www.prisonstudies.org/sites/default/files/resources/downloads/world_prison_population_list_14th_edition.pdf

[aot70042-bib-0023] Farnworth, L. , & Muñoz, J. P. (2009). An occupational and rehabilitation perspective for institutional practice. Psychiatric Rehabilitation Journal, 32, 192–198. 10.2975/32.3.2009.192.198 19136351

[aot70042-bib-0024] Ford, E. , Di Tommaso, A. , Molineux, M. , & Gustafsson, L. (2022). Identifying the characteristics of occupation‐centred practice: A Delphi study. Australian Occupational Therapy Journal, 69(1), 25–37. 10.1111/1440-1630.12765 34490901

[aot70042-bib-0025] Gentry, K. K. Jr. , Snyder, K. , & Utley, J. J. (2021). Clinical utility of the adapted biopsychosocial model: An initial validation through peer review. Open Journal of Occupational Therapy, 9(2), 1–20. 10.15453/2168-6408.1750

[aot70042-bib-0026] Gonzalez, A. V. , Eikenberry, J. , Griess, C. , Jaegers, L. , & Baum, C. M. (2023). Evaluation of an occupational therapy reentry program: Achieving goals to support employment and community living after incarceration. Work, 75(2), 639–656. 10.3233/WOR-220035 36641720

[aot70042-bib-0027] Haesen, S. , Merkt, H. , Imber, A. , Elger, B. , & Wangmo, T. (2019). Substance use and other mental health disorders among older prisoners. International Journal of Law and Psychiatry, 62, 20–31. 10.1016/j.ijlp.2018.10.004 30616851

[aot70042-bib-0028] Hare Duke, L. , Furtado, V. , Guo, B. , & Völlm, B. A. (2018). Long‐stay in forensic‐psychiatric care in the UK. Social Psychiatry and Psychiatric Epidemiology, 53(3), 313–321. 10.1007/s00127-017-1473-y 29387921 PMC5842247

[aot70042-bib-0029] Hong, Q. N. , Pluye, P. , Fàbregues, S. , Bartlett, G. , Boardman, F. , Cargo, M. , Dagenais, P. , Gagnon, M.‐P. , Griffiths, F. , & Nicolau, B. (2019). Improving the content validity of the mixed methods appraisal tool: A modified e‐Delphi study. Journal of Clinical Epidemiology, 111, e41. 10.1016/j.jclinepi.2019.03.008 30905698

[aot70042-bib-0030] Huang, A. , Vrklevski, L. , McGregor, F. , & Yu, L. (2023). Patient outcomes in an Australian low secure forensic psychiatric rehabilitation inpatient unit: A 10‐year retrospective study. Psychiatry, Psychology and Law, 30(4), 486–500. 10.1080/13218719.2022.2059026 PMC10360983 37484506

[aot70042-bib-0031] Ismail, N. , & de Viggiani, N. (2017). Should we use a direct regulation to implement the Healthy Prisons Agenda in England? A qualitative study among prison key policy makers. Journal of Public Health, 40(3), 598–605. 10.1093/pubmed/fdx116 28977435

[aot70042-bib-0032] Jaegers, L. A. , Skinner, E. , Conners, B. , Hayes, C. , West‐Bruce, S. , Vaughn, M. G. , Smith, D. L. , & Barney, K. F. (2020). Evaluation of the jail‐based occupational therapy transition and integration services program for community reentry. American Journal of Occupational Therapy, 74(3), 1–11. 10.5014/ajot.2020.035287 32365309

[aot70042-bib-0033] Jeker, B. , Shaw, D. , Lagnaux, N. , Wangmo, T. , & Elger, B. S. (2023). Motivation and training needs of prison healthcare professionals: Findings from a qualitative study. BMC Psychology, 11(1), 167. 10.1186/s40359-023-01076-8 37210567 PMC10199499

[aot70042-bib-0034] Kang‐Brown, J. , Montagnet, C. , & Heiss, J. (2021). People in Jail and Prison in Spring 2021. Vera Institute of Justice. https://www.vera.org/publications/people‐in‐jail‐and‐prison‐in‐spring‐2021, https://pdfs.semanticscholar.org/13fa/1a67e4f1989a05f600fc9aaf64e306f851d0.pdf

[aot70042-bib-0035] Karaaslan, A. , & Aslan, M. (2019). The Relationship Between the Quality of Work and Organizational Commitment of Prison Nurses. Journal of Nursing Research, 27(3), e25. 10.1097/jnr.0000000000000286 PMC6553965 30239374

[aot70042-bib-0036] Kennedy, H. G. , Simpson, A. , & Haque, Q. (2019). Perspective on excellence in forensic mental health services: What we can learn from oncology and other medical services. Frontiers in Psychiatry, 10, 733. 10.3389/fpsyt.2019.00733 31681042 PMC6813277

[aot70042-bib-0037] Kinner S. , & Butler T. (2017). Public Health Association of Australia: Prisoner health background paper Public Health Association Australia.

[aot70042-bib-0038] Kyprianides, A. , & Easterbrook, M. J. (2020). Social Factors Boost Well‐Being Behind Bars: The Importance of Individual and Group Ties for Prisoner Well‐Being. Applied Psychology: Health and Well‐Being, 12(1), 7–29. 10.1111/aphw.12171 31215172

[aot70042-bib-0039] Leonard, S. (2004). The successes and challenges of developing a prison telepsychiatry service. Journal of Telemedicine and Telecare, 10(1_suppl), 69–71. 10.1258/1357633042614375 15603615

[aot70042-bib-0040] Liberati, A. , Altman, D. G. , Tetzlaff, J. , Mulrow, C. , Gøtzsche, P. C. , Ioannidis, J. P. , Clarke, M. , Devereaux, P. J. , Kleijnen, J. , & Moher, D. (2009). The PRISMA statement for reporting systematic reviews and meta‐analyses of studies that evaluate health care interventions: Explanation and elaboration. Journal of Clinical Epidemiology, 62(10), e1–e34. 10.1016/j.jclinepi.2009.06.006 19631507

[aot70042-bib-0041] Maruca, A. T. , & Shelton, D. (2016). Correctional Nursing Interventions for Incarcerated Persons with Mental Disorders: An Integrative Review. Issues in Mental Health Nursing, 37(5), 285–292. 10.3109/01612840.2016.1145308 27049171

[aot70042-bib-0042] McKenna, B. , & Sweetman, L. E. (2021). Models of care in forensic mental health services: A review of the international and national literature. Ministry of Health.

[aot70042-bib-0043] McLeod, K. E. , Butler, A. , Young, J. T. , Southalan, L. , Borschmann, R. , Sturup‐Toft, S. , Dirkzwager, A. , Dolan, K. , Acheampong, L. K. , Topp, S. M. , Martin, R. E. , & Kinner, S. A. (2020). Global prison health care governance and health equity: A critical lack of evidence. American Journal of Public Health, 110(3), 303–308. 10.2105/ajph.2019.305465 31944844 PMC7002953

[aot70042-bib-0044] Mir, J. , Kastner, S. , Priebe, S. , Konrad, N. , Ströhle, A. , & Mundt, A. P. (2015). Treating substance abuse is not enough: Comorbidities in consecutively admitted female prisoners. Addictive Behaviors, 46, 25–30. 10.1016/j.addbeh.2015.02.016 25770695

[aot70042-bib-0045] Mullen, A. , Browne, G. , Hamilton, B. , Skinner, S. , & Happell, B. (2022). Safewards: An integrative review of the literature within inpatient and forensic mental health units. International Journal of Mental Health Nursing, 31(5), 1090–1108. 10.1111/inm.13001 35365947 PMC9544259

[aot70042-bib-0046] Muñoz, J. P. , Moreton, E. M. , & Sitterly, A. M. (2016). The Scope of Practice of Occupational Therapy in U.S. Criminal Justice Settings. Occupational Therapy International, 23(3), 241–254. 10.1002/oti.1427 27094024

[aot70042-bib-0047] Mynard, L. , Joosten, A. , D'Souza, A. , Ashley, D. , & Darzins, S. (2024). Occupational therapy with patients in forensic solitary confinement: A qualitative study. Australian Occupational Therapy Journal, 71(4), 447–460. 10.1111/1440-1630.12930 38253942

[aot70042-bib-0048] Nishar, S. , Brumfield, E. , Mandal, S. , Vanjani, R. , & Soske, J. (2023). “It's a revolving door”: understanding the social determinants of mental health as experienced by formerly incarcerated people. Health & Justice, 11(1), 26. 10.1186/s40352-023-00227-8 37300627 PMC10256957

[aot70042-bib-0049] Peters, M. D. , Marnie, C. , Tricco, A. C. , Pollock, D. , Munn, Z. , Alexander, L. , McInerney, P. , Godfrey, C. M. , & Khalil, H. (2020). Updated methodological guidance for the conduct of scoping reviews. JBI Evidence Synthesis, 18(10), 2119–2126. 10.11124/JBIES-20-00167 33038124

[aot70042-bib-0051] Piacentini, L. , Weaver, B. , & Jardine, C. 2018 Employment and Employability in Scottish Prisons: A Research Briefing Paper

[aot70042-bib-0052] Piquero, A. R. , & Rocque, M. (2020). Changing self‐control: Promising efforts and a way forward. New Directions for Child and Adolescent Development, 2020(173), 39–47. 10.1002/cad.20368 33029851

[aot70042-bib-0053] Pont, J. , & Harding, T. W. (2019). Organisation and management of health care in prison. Council of Europe.

[aot70042-bib-0054] Pont, J. , Stöver, H. , & Wolff, H. (2012). Dual loyalty in prison health care. American Journal of Public Health, 102(3), 475–480. 10.2105/AJPH.2011.300374 22390510 PMC3487660

[aot70042-bib-0055] Queensland Health and Queensland Corrective Service . (2020). Reducing barriers to health and wellbeing: The Queensland Prisoner Health and Wellbeing Strategy 2020–2025

[aot70042-bib-0056] Ramakers, A. , Nieuwbeerta, P. , Van Wilsem, J. , & Dirkzwager, A. (2017). Not Just Any Job Will Do: A Study on Employment Characteristics and Recidivism Risks After Release. International Journal of Offender Therapy and Comparative Criminology, 61(16), 1795–1818. 10.1177/0306624X16636141 26975405 PMC5669259

[aot70042-bib-0057] Ronco, D. (2021). Health protection in prison, between equivalence of care and less eligibility. Research on Humanities and Social Sciences, 11(11), 65–71.

[aot70042-bib-0058] Shea, C. K. , & Siu, A. M. (2016). Engagement in play activities as a means for youth in detention to acquire life skills. Occupational Therapy International, 23(3), 276–286. 10.1002/oti.1432 27363848

[aot70042-bib-0059] Simon, L. , Beckmann, D. , Stone, M. , Williams, R. , Cohen, M. , & Tobey, M. (2020). Clinician experiences of care provision in the correctional setting: A scoping review. Journal of Correctional Health Care, 26(4), 301–314. 10.1177/1078345820953154 32873120

[aot70042-bib-0060] Sridhar, S. , Cornish, R. , & Fazel, S. (2018). The Costs of Healthcare in Prison and Custody: Systematic Review of Current Estimates and Proposed Guidelines for Future Reporting. Frontiers in Psychiatry, 9, 716. 10.3389/fpsyt.2018.00716 30618885 PMC6306428

[aot70042-bib-0061] Sturge, G. (2021). UK Prison Population Statistics. House of Commons.

[aot70042-bib-0062] Tadros, E. , Barbini, M. , & Kaur, L. (2023). Collaborative healthcare in incarcerated settings. International Journal of Offender Therapy and Comparative Criminology, 67(9), 910–929. 10.1177/0306624X211058952 34784803

[aot70042-bib-0063] Tan, B. L. , Ravindra Kumar, V. , & Devaraj, P. (2015). Development of a new occupational therapy service in a Singapore prison. British Journal of Occupational Therapy, 78(8), 525–529. 10.1177/030802261557108

[aot70042-bib-0064] Tavoschi, L. , O'Moore, É. , & Hedrich, D. (2019). Challenges and opportunities for the management of infectious diseases in Europes' prisons: evidence‐based guidance. Lancet Infectious Diseases, 19(7), e253–e258. 10.1016/S1473-3099(18)30756-4 30902441

[aot70042-bib-0065] Tomlin, J. , Pham, T. , Lega, I. , Braun, P. , Kennedy, H. G. , Herrando, V. T. , Barroso, R. , Castelletti, L. , Mirabella, F. , Scarpa, F. , Völlm, B. , Müller‐Isberner, R. , Taube, M. , Rivellini, G. , Calevro, V. , Liardo, R. , Pennino, M. , Markiewicz, I. , & Barbosa, F. (2021). Forensic mental health in Europe: Some key figures. Social Psychiatry and Psychiatric Epidemiology, 56(1), 109–117. 10.1007/s00127-020-01909-6 32651594 PMC7847441

[aot70042-bib-0066] Tricco, A. C. , Lillie, E. , Zarin, W. , O'Brien, K. K. , Colquhoun, H. , Levac, D. , Moher, D. , Peters, M. D. J. , Horsley, T. , Weeks, L. , Hempel, S. , Akl, E. A. , Chang, C. , McGowan, J. , Stewart, L. , Hartling, L. , Aldcroft, A. , Wilson, M. G. , Garritty, C. , … Straus, S. E. (2018). PRISMA extension for scoping reviews (PRISMA‐ScR): Checklist and explanation. Annals of Internal Medicine, 169(7), 467–473. 10.7326/m18-0850 30178033

[aot70042-bib-0067] Tully, J. , Hafferty, J. , Whiting, D. , Dean, K. , & Fazel, S. (2024). Forensic mental health: envisioning a more empirical future. Lancet Psychiatry, 11(11), 934–942. 10.1016/S2215-0366(24)00164-0 38945145

[aot70042-bib-0068] U.S. Department of Health and Human Services . (2020). Incarceration. https://health.gov/healthypeople/priority-areas/social-determinants-health/literature-summaries/incarceration

[aot70042-bib-0069] Vandala, N. G. (2019). The transformative effect of correctional education: A global perspective. Cogent Social Sciences, 5(1), 1677122. 10.1080/23311886.2019.1677122

[aot70042-bib-0070] Wardrop, R. , Ranse, J. , Chaboyer, W. , & Crilly, J. (2021). Structures, processes and outcomes of health care for people detained in short‐term police custody settings: A scoping review. Journal of Forensic Legal Medicine, 81, 102198. 10.1016/j.jflm.2021.102198 34147830

[aot70042-bib-0071] Whiteford, G. , Jones, K. , Weekes, G. , Ndlovu, N. , Long, C. , Perkes, D. , & Brindle, S. (2020). Combatting occupational deprivation and advancing occupational justice in institutional settings: Using a practice‐based enquiry approach for service transformation. British Journal of Occupational Therapy, 83(1), 52–61. 10.1177/030802261986522

[aot70042-bib-0072] Wong, I. , Wright, E. , Santomauro, D. , How, R. , Leary, C. , & Harris, M. (2018). Implementing two nurse practitioner models of service at an Australian male prison: A quality assurance study. Journal of Clinical Nursing, 27(1–2), e287–e300. 10.1111/jocn.13935 28639389

[aot70042-bib-0073] Woodall, J. , De Viggiani, N. , & South, J. (2022). Salutogenesis in Prison (pp. 553–561). Springer International Publishing. 10.1007/978-3-030-79515-3_51 36121978

[aot70042-bib-0074] World Federation of Occupational Therapists . (2020). World Federation of Occupational Therapists. https://www.wfot.org/

[aot70042-bib-0075] Yoon, I. A. , Slade, K. , & Fazel, S. (2017). Outcomes of psychological therapies for prisoners with mental health problems: A systematic review and meta‐analysis. Journal of Consulting and Clinical Psychology, 85(8), 783–802. 10.1037/ccp0000214 28569518 PMC5518650

[aot70042-bib-0076] Young, J. T. , Heffernan, E. , Borschmann, R. , Ogloff, J. R. P. , Spittal, M. J. , Kouyoumdjian, F. G. , Preen, D. B. , Butler, A. , Brophy, L. , Crilly, J. , & Kinner, S. A. (2018). Dual diagnosis of mental illness and substance use disorder and injury in adults recently released from prison: a prospective cohort study. Lancet. Public Health, 3(5), e237–e248. 10.1016/S2468-2667(18)30052-5 29680329

[aot70042-bib-0077] Yukhnenko, D. , Sridhar, S. , & Fazel, S. (2019). A systematic review of criminal recidivism rates worldwide: 3‐year update. Wellcome Open Research, 4, 28. 10.12688/wellcomeopenres.14970.3 31544154 PMC6743246

[aot70042-bib-0078] Zarling, A. , Scheffert, R. , & Russell, D. (2021). Predictors of retention and recidivism of justice‐involved women in a community‐based gender‐responsive CBT program. Criminal Justice and Behavior, 49(3), 291–310. 10.1177/0093854821104085

