# Supplementary material for: The role, challenges, and impact of occupational therapists in custodial settings: A scoping review
Source: Aust Occup Ther J. 2025 Aug 7;72(4):e70042. doi: 10.1111/1440-1630.70042 (PMC12332472; doi:10.1111/1440-1630.70042)
Supplement: Supplementary file 1 — Table S1. Search strategy of CINAHL database. [file AOT-72-e70042-s001.docx]

**SUPPLEMENTARY FILE TABLE S1. Search strategy of CINAHL database**

| ***No.*** | ***Search strategy*** | ***Number of articles retrieved*** |
| --- | --- | --- |
| S11 | S8 AND S9 + Date limiter 2013 -2023 & English language only | 165 |
| S10 | S8 AND S9 | 274 |
| S9 | S2 OR S5 OR S6 OR S7 | 40,185 |
| S8 | S1 OR S3 OR S4 | 53,747 |
| S7 | (MH "Prisoners") OR Inmate OR Convict* | 25,027 |
| S6 | (MH "Correctional Health Services") | 1,921 |
| S5 | (MH "Correctional Facilities") | 7,284 |
| S4 | (MH "Occupational Therapists") | 10,025 |
| S3 | (MH "occupational therapy") OR (MH "Occupational Therapy Assistants") | 24,277 |
| S2 | custodial* OR Prison* OR Incarcerat* OR Jail* OR "Correction* Facilities" OR "Forensic psychiatric hospital" OR detention OR "Watch house" OR Custody* OR gaol* OR penitentiary | 26,807 |
| S1 | "occupational therap*" OR OT OR OTA | 53,747 |
